# Endoscopic Nipple- or Skin-Sparing Mastectomy and Immediate Breast Reconstruction with Endoscopic Harvesting of the Latissimus Dorsi Flap: A Preliminary Experience of an Innovative Technique

**DOI:** 10.1155/2022/1373899

**Published:** 2022-10-27

**Authors:** Yu Feng, Nan Wen, Faqing Liang, Jiao Zhou, Xiangquan Qin, Xinran Liu, Juan Li, Mengxue Qiu, Huanzuo Yang, Zhenggui Du

**Affiliations:** ^1^Department of Breast Surgery, West China Hospital, Sichuan University, Chengdu 610041, Sichua, China; ^2^Department of Breast Surgery, Sichuan Academy of Medical Sciences, Sichuan Province People's Hospital, Chengdu 610072, Sichuan, China; ^3^Breast Disease Research Center, West China Hospital, Sichuan University, Chengdu 610041, Sichuan, China

## Abstract

**Background:**

Endoscopic nipple- or skin-sparing mastectomy (E-N/SSM) and endoscopic latissimus dorsi muscle flap (E-LDMF) harvest have been operational difficulties over decades. The aim of this study was to describe the preliminary outcomes of our novel surgical technique, which allows the performance of E-N/SSM and E-LDMF harvest for immediate breast reconstruction (IBR) through a single cosmetic axillary incision for breast cancer patients.

**Methods:**

This prospective study included 20 breast cancer patients who underwent E-N/SSM and E-LDMF harvesting through a single axillary incision in our hospital from September 2020 to June 2022. The outcomes were statistically calculated, including patient characteristics, operative data, complication rate, hospital length of stay and costs, and patient-reported outcomes.

**Results:**

A total of 20 breast cancer patients underwent our sufficiently mature novel endoscopy technique. The mean LD flap harvest time was 96.5 ± 25.3 min, the mean operation time was 262.6 ± 54.4 min, and the average length of LDMF was 26.9 ± 3.1. During the median follow-up time of 7.5 months, 4 patients developed donor-site seroma. One of them was also complicated by hypopigmentation of the nipple areola, and one of them suffered from breast cellulitis. No bleeding or flap necrosis happened. No tumor recurrence or metastasis was found until the last follow-up. In the BREAST-Q evaluation, although they gave a lower score beginning at 1-month post-operation than preoperatively (*P* > 0.05, except for physical well-being: chest and physical well-being: back and shoulder, *P* < 0.01), there was an uptrend at 3 months postoperatively. Because of the hidden and short incision, the mean score of the appearance scale of the SCAR-Q at 3 months post-operation was 74.2 ± 8.8.

**Conclusions:**

The novel endoscopy technique, which was first reported to perform lymph node surgery, N/SSM, and LDMF harvesting in an operation for breast cancer patients through a single axillary incision, is associated with a shorter surgery time, lower complication rates, and better patient-reported outcomes.

## 1. Introduction

Statistics from the National Cancer Center of China show that the incidence of breast cancer in China was approximately 272,400 cases per year in 2015, with higher incidences in developed regions and a trend of rejuvenation [1]. With the advancement of breast cancer systemic treatment and surgical technology, the 5-year survival rate of breast cancer in China rose from 75.9% in 2000–2004 to 83.2% in 2010–2014, and there is still room for further improvement [2]. For breast cancer patients with such a high postoperative survival rate, especially the younger population, saving lives is no longer the only purpose of treatment, but how to protect or restore the shape of the breast and improve patient quality of life (QoL) to the greatest extent while undergoing treatment to further promote their social return is particularly important. In China, the proportion of breast cancer patients receiving total mastectomy is as high as 81.3% [3], so breast reconstruction is an important way to ensure the appearance of the breasts. Compared with alloplastic breast reconstruction, autologous tissue (flap) can help restore a perfect shape of the breast by providing muscle coverage on the surface of the prosthesis, replacing the skin of the breast, and replacing the gland as a volume replacement technique, creating a breast with a perfect shape, long-term durability and touch, and a natural sagging degree [4, 5]. Among the autologous tissues, LDMF is the one with the best operability and high survival rate due to its reliable vascular pedicle, adequate blood supply, and wide dimensions and has been spotlighted as a workhorse flap in reconstruction surgery [5–8]. In patients who have undergone or are estimated to carry on radiotherapy, LDMF is better tolerated than the prosthesis, and it has less fat than the abdominal flap, with fewer side effects of radiotherapy [5, 9]. The LDMF provides better prosthetic coverage than mesh-combined prosthetic breast reconstruction and greatly reduces the rate of postoperative infection, prosthetic exposure, and removal. However, the open harvesting technique of LD will leave a 15–45 cm scar on the back, which is unacceptable for most patients [6, 10, 11].

To reduce or even eliminate the incisions and scars on the breasts and back, endoscopic and robotic technologies have been used, but neither procedure has been widely used. There were difficulties with traditional E-LDMF harvesting, such as long and straight laparoscopic surgical instruments that cannot be turned around the curved contour of the back, difficulty in reaching the edge of the LD, difficulty in maintaining good vision, and disfluency manipulation, making it stressful and surgically challenging, with a prolonged operation time and insufficient capacity of LD [10, 12], thus limiting its application in breast reconstruction. Although robotics improves visibility and flexibility, intraoperative changes in lens positions are relatively cumbersome and less convenient than endoscopic changes. Furthermore, robotic surgery requires more expensive instruments, longer operation times, and long learning curves [10, 13–15], making it difficult to perform routine surgery. In addition, according to the literature, some endoscopic or robotic-assisted breast reconstructions may require multiple incisions or ports, and scar concealment is poor [8, 10, 16].

After 2 years of exploration, the Department of Breast Surgery of West China Hospital has created its own simple and flexible axillary endoscopic technology, which has changed the order of routine LDMF harvesting. Combined with the auxiliary holes on the back and breast, this novel technique steeply reduced the surgical difficulty and quickly finished axillary surgery, E-N/SSM, E-LDMF harvesting, and IBR in one surgery through a single concealed axillary incision [17]. This has made it possible to perform the procedure in a wider range of hospitals to treat breast cancer patients. The aim of this study was to describe the preliminary outcomes of our novel surgical technique, which allows the performance of E-N/SSM and E-LDMF harvest followed by immediate breast reconstruction (IBR) through a single cosmetic axillary incision for breast cancer patients.

## 2. Patients and Methods

### 2.1. Patients

A total of 20 breast cancer patients who simultaneously underwent IBR following E-N/SSM and endoscopic harvesting LDMF for breast cancer in West China Hospital from September 2020 to June 2022 were enrolled in this prospective study. Surgical procedures of 20 patients, including axillary surgery, E-LDMF harvesting, E-N/SSM, and IBR, were conducted by one surgeon during the study. Informed consent was obtained from all patients. The number of cases in this institution during the study period determined the sample size.

Inclusion criteria include (1) multicentric breast carcinoma and large unicentric carcinomas (<5 cm) that would not be suitable for breast-conserving surgery; (2) large (>5 cm) unicentric carcinoma localized in the mammary gland shrinks to less than 5 cm after neoadjuvant chemotherapy; (2) no chest wall, skin, or nipple-areolar complex (NAC) tumor invasion (including Paget's disease); (3) mild to moderate breast ptosis with a desire for reconstruction and refusal of meshes; (4) thin subcutaneous fat; (5) patients who have undergone preoperative radiotherapy or who may require radiotherapy postoperatively; (6) high requirements for postoperative breast shape and feel; and (7) LD muscle combined prosthesis reconstruction is indicated for patients with insufficient volume of LDMF.

Contraindications include (1) inflammatory breast cancer; (2) distant metastasis of the tumor; (3) history of vascular injury to the axillary thoracic dorsum; and (4) severe comorbid conditions.

### 2.2. Novel Surgical Techniques

#### 2.2.1. Preoperative Marking

**Figure 1:**
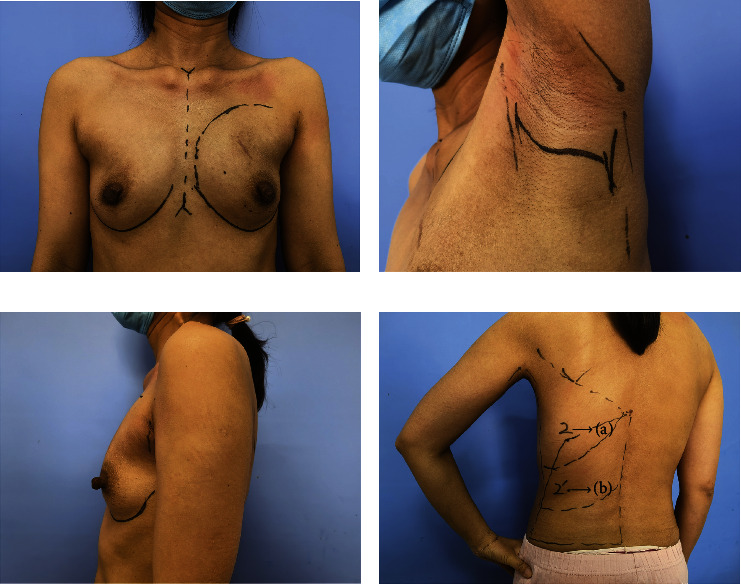
Preoperative marking. (a) Breast marking; (b) marking of the axillary incision line of approximately 4-5 cm; (c) complete coverage of the incision by the sagged arm; (d) marking of the borders of the LD. (i) “Huaxi hole 2” is a manmade 0.5 cm incision located 2-3 cm inner to the posterior axillary line at a distance of 15–20 cm from the top of the axilla in the posterior and (ii) “Huaxi hole 2′” is determined by the required volume of LD muscle. Fan chart with around 15 cm radius with the axillary incision as the center represents the excision range from the axillary incision and the fan chart with around 15 cm radius with the “Huaxi hole 2” as the center represents the excision range from the “Huaxi hole 2.”

Preoperative marking is illustrated in Figure 1. The patient was placed in a seated position, and a solid line was drawn at the inframammary fold, medial margin, and lateral margin of the breast. An embedded-in-axilla incision line of approximately 4-5 cm was drawn, which could be completely covered by her arm when it sags naturally. A 5 mm skin incision (named “Huaxi hole 1” and allowed electric knife access) was marked, located next to the areola in the upper-outer quadrant for N/SSM. The borders of the LD along the posterior axillary line, inferior margin at the iliac crest, medial margin along the paravertebral origin, and superior margin at the tip of the scapula were marked. A small 5 mm incision (2-3 cm inner to the posterior axillary line at a distance of 15–20 cm from the top of the axilla) was marked as a hole (“Huaxi hole 2”) for operation and the outlet for postoperative drainage tube placement. If more volume of LD muscle was needed, “Huaxi hole 2' ” can be added (the position is determined by the volume of LD muscle).

After general anesthesia, the patient was primarily positioned in the lateral recumbent position with the operative side uppermost and was pad fixed for the buttock and shoulder, which can reduce intraoperative position change and thus save operative time. The operated side arm should be wrapped particularly to lift it to the forehead during surgery and expose the axillary fossa under endoscopy. A detailed video demonstrating the procedure can be accessed in Video 1 (https://drive.google.com/file/d/1GkViAvhysNhsj0j18b79S5rGgBeFd10m/view?usp=sharing).

#### 2.2.2. Axillary Surgery

Sentinel lymph node biopsy (SLNB) is primarily performed under direct visualization through the axillary incision. If preoperative aspiration biopsy or SLNB was positive, axillary lymph node dissection (ALND) was recommended.

#### 2.2.3. LDMF Harvesting

**Figure 2:**
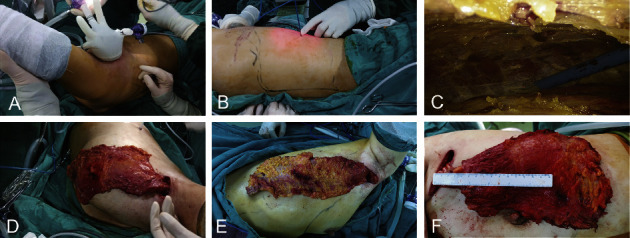
LDMF harvesting. (a) Self-made access with an 80 mm disposable wound protector wrapped by the opening end of one sterile surgical glove of the right hand (^#^7.5); (b-c) insert the electric knife through “Huaxi hole 2”; (d–f) different sizes of harvested LD (the surrounding fat fascia tissue of LDMF was taken together for some patients to ensure that there was enough tissue volume to meet the needs of breast filling).

The vessel branch to the anterior serratus muscle of the thoracodorsal vessels was transected under direct vision through the axillary incision to avoid the limitation of LDMF rotation and bulge of axillary, taking care to protect the thoracodorsal vessels and accompanying nerves in the process. LDMF was dissected with a reverse dissection order (from deep to superficial planes). The anterolateral part of the LD muscle was dissected first under direct visualization, with its superior margin reaching to the lower edge of the subscapularis muscle, taking care not to dissect the deep surface of the subscapularis muscle. Continue dissociating LD toward the spine and the iliac bone until difficulties are encountered with direct visualization. An 80 mm disposable wound protector wrapped by the opening end of one sterile surgical glove of the right hand (^#^7.5) was placed through the incision. Two trocars (12.5 mm and 5.5 mm) were inserted into different finger holes of the glove to create entry sites for the instruments (Figure 2(a)). CO_2_ gas insufflation was performed at 12 mm·Hg (flow rate of 20–40 L/min) to create and maintain patency and sufficient optical cavity tension. An electric knife and coagulation hook were used to continue the separation of the flap to a point where it cannot reach. The electric knife was inserted directly into the submuscular plane through “Huaxi hole 2” (a manmade 0.5 cm incision located 2-3 cm inner to the posterior axillary line at a distance of 15–20 cm from the top of the axilla) (Figures 2(b) and 2(c)). With the help of gas insufflation and a gripper, the electric knife acts as a “relay baton” that continues dissecting the remaining LD until the marked border under the endoscope and cuts the LD muscle. If more LDMF was needed, a “Huaxi hole 2' ” was made to continue the separation of the flap until the desired range of LD muscle for cutting. The subcutaneous layer was similarly dissected. The appropriate thickness of subcutaneous fat can be retained on the surface of the LD depending on the size of the tissue required for breast reconstruction (Figures 2(d)–2(f)). The dissection order of the submuscle is lateral margin, superior margin, paravertebral origin, and inferior margin. However, it is important to note that subcutaneous layer dissection should be carried out in the order of the superior margin, the paravertebral origin, the inferior margin, and the lateral margin. And the ultrasonic knife was used to stop the bleeding.

#### 2.2.4. Breast Surgery

The excised LD muscle was placed in a subcutaneous pocket in the axilla, and negative suction drains were inserted via “Huaxi hole 2” and “Huaxi hole 2' ”. The lateral position was changed to a supine position by removing the pads supporting the buttock and shoulder without the need for disinfection repreparation for the surgical field again. Then, E-N/SSM was performed in the order of the retro mammary space (by electric knife and a coagulation hook) and the subcutaneous layer of the breast, which was dissected using the electric knife to retro-areolar tissue through the axillary incision, and the rest of the layer was then performed using the electric knife inserted through the “HUAXI hole 1” (located next to the areola in the upper-outer quadrant). The surgical procedure was detailed in primary studies [18, 19].

#### 2.2.5. Reconstructive Surgery

If LDMF combined with prosthesis breast reconstruction was performed, LDMF would move into the subcutaneous cavity of the breast. The upper or lateral border of the LD muscle is sutured to the inferior border of the anterior serratus and fascia to form the lateral wall of the implant cavity to avoid outward migration of the implant. The prosthesis was placed between the deep surface of the LD muscle and the superficial surface of the pectoralis major muscle, and the position of the implant was adjusted to be bilaterally symmetrical. If LDMF alone was used for breast reconstruction, the lateral edge of the LD muscle was fixed to the upper edge of the subcutaneous tissue of the breast with appropriate interrupted sutures, and the LD muscle was adjusted with a naturally drooping position and shape to achieve bilateral symmetry. There is no need to redefine the inframammary fold or fix the LD to the inframammary fold. As the LD muscle shrinks over time, the reconstructed side of the breast is generally 50% larger than the non-operated side.

#### 2.2.6. Postoperative Management

A postoperative pressure dressing was required routinely over the back, and negative pressure was continuously used to induce drainage and prevent the formation of seroma of the back. The drainage tube of the back is usually removed when the flow is less than 20 ml/24 h for 3 consecutive days, which is stricter than the drain removal criteria of the breast (less than 30 ml per day for 3 consecutive days). The patient was placed in a semisitting position for 2 hours after surgery, relying on the weight of the prosthesis and the LD muscle to create a natural droop, and was required to wear a breast contouring garment for 3 months with appropriate tightness to avoid ischemia and necrosis of the skin and muscle flap.

#### 2.2.7. Outcome Measures Evaluation

Demographic information collected from the patients included age, body mass index (BMI), grade of breast ptosis, cup size, tumor location, breast cancer stage, mode of axillary surgery, weight of the excised gland, total operation time, E-LDMF harvesting time, E-N/SSM time, length of hospital stays, hospitalization expenses, complications, and pathology. Patients in the study received the BREAST-Q™ Reconstruction Module questionnaire [20] preoperatively and 1 month and 3 months postoperatively to evaluate QoL (psychosocial well-being, sexual well-being, physical well-being: chest, physical well-being: back and shoulder) and patient-reported aesthetic results (satisfaction with breast and back). All patients assessed their own postoperative scars using the SCAR-Q questionnaire at 3 months post-operation. The final score was transformed on a scale of 0–100 according to the BREAST-Q or SCAR-Q protocol, with higher scores equating to higher satisfaction. Photographs of the patient's breasts and backs were taken and shown to them for self-evaluation.

Postoperative complications, including surgical site infection (SSI), bleeding, wound dehiscence, breast/donor-site skin flap/NAC necrosis, seroma of chest and donor-site, lymphedema of surgical side, implant rupture, migration or removal, and capsular contracture, were collected in this study, of which complications requiring unplanned reoperation, intravenous antibiotics, or readmission were defined as major complications. The recurrence and survival status of patients were recorded at the last follow-up. Seroma is any fluid that accumulates under the skin flap or in the axillary cavity after mastectomy and requires clinical aspiration or image-guided aspiration, according to the criteria for the determination of seroma by Marquez [21] and Srivastava [22].

### 2.3. Statistical Analysis

We used SPSS 25 for Windows (SPSS Inc., Chicago, IL) for statistical analyses and R programming language (version 4.0.2, R Development Core Team 2020) for mapping. Continuous data are summarized as the mean ± standard deviation, and Student's *t*-test or the Wilcoxon signed-rank test was used for comparisons. Categorical variables are presented as frequencies and proportions, and the chi-square test or Fisher's exact test was used for analysis. All *P* values were two-tailed, and a *P* value <0.05 was considered statistically significant.

## 3. Results

### 3.1. Clinical and Pathological Characteristics

A total of 20 breast cancer patients, with a mean BMI of 21.3 ± 2.7 kg/m^2^, underwent the novel technique of E-N/SSM and E-LDMF harvesting for IBR through a single axillary incision and were prospectively included in this study. All patients underwent unilateral surgery, with 9 patients having right breasts and 11 patients having left breasts. Invasive breast cancer (50%) accounted for the largest proportion of the 20 patients in our study. All patients had completed the follow-up. Demographic data are reported in Table 1.

### 3.2. Operative and Perioperative Data

**Figure 3:**
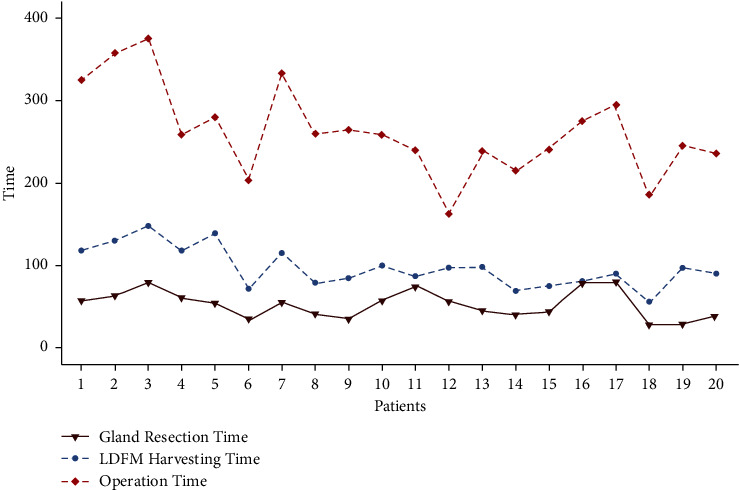
The changing trend of the operation-related time of the 20 patients.

All the patients successfully completed this novel endoscopic surgery with no case of intraoperative conversion to open surgery. The mean mastectomy weight was 278.3 ± 82.3 g. All patients obtained negative margins. It was identified that it took more time in the initial period, but the operating time was reduced from 375 min to 163 min as process optimization, repetition, and accumulation occurred, during which the E-LDMF harvesting time decreased from 148 min to 56 min (Figure 3). With the assistance of “Huaxi hole 2” and “Huaxi hole 2' ”, we can obtain more LDMF, with a length of 26.9 ± 3.1 (20 to 34) cm and a width of 13.8 ± 2.1 (11–18) cm. The total hospitalization costs of LDMF and LDMF combined prosthesis breast reconstruction were 3401.6 and 5434.8 USD, respectively (*P* < 0.01). The operative and perioperative data are shown in Table 2.

### 3.3. Complications

During a median follow-up of 7.5 months, 4 patients developed donor-site seroma, and all were treated with the placement of a drain in the office. One of the three patients was also complicated by hypopigmentation of nipple areola and was given observation, and one of them who underwent LDMF and prosthesis breast reconstruction suffered breast cellulitis with oral antibiotics treatment (minor complication rate: 20%). No other complications such as bleeding, ischemic necrosis of the breast skin flap, donor-site skin or NAC, lymphedema of the surgical side, chest seroma, or implant loss were observed.

### 3.4. Aesthetic Results and QoL

**Figure 4:**
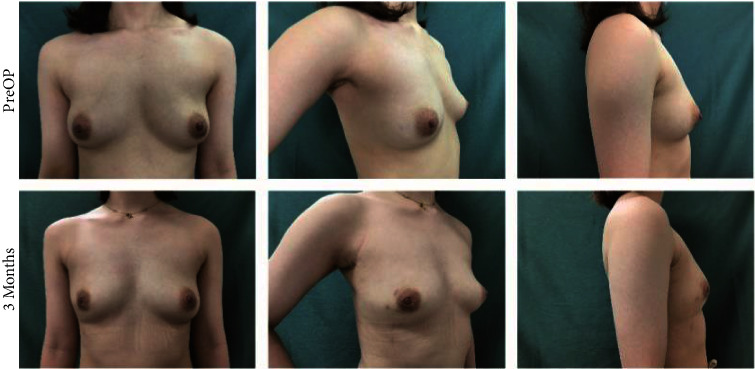
Photographs of this 30-year-old woman show the preoperative view and 3 months after immediate endoscopic mini LD harvest for volume replacement after an endoscopic partial mastectomy, with a mastectomy weight of 180 g, length of 20 cm, and width of 13 cm of LDMF.

**Figure 5:**
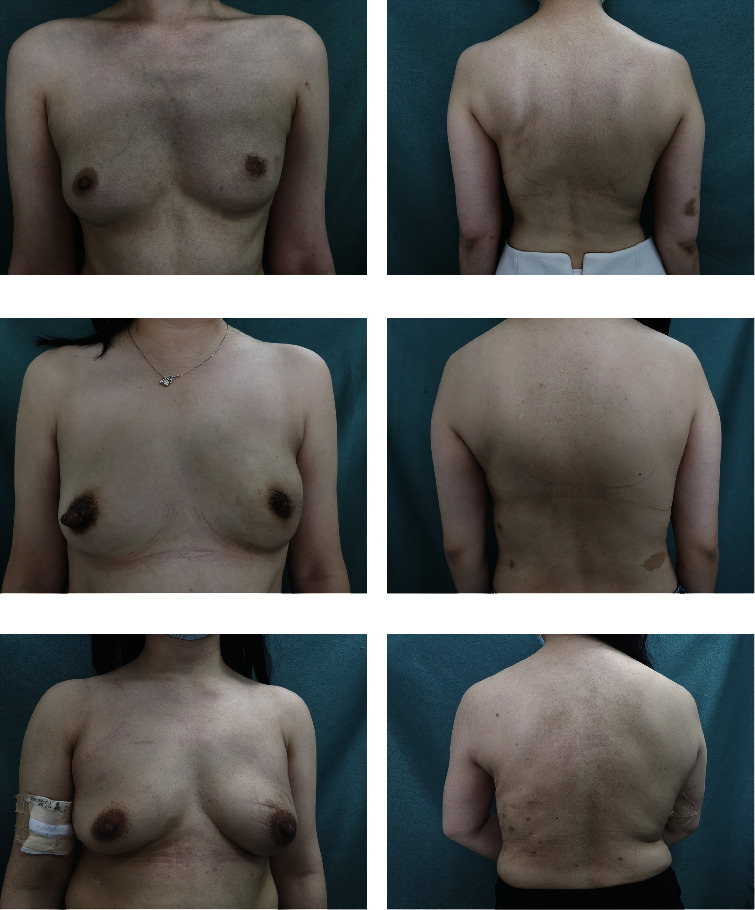
(a-b) Photos of a 34-year-old woman with her left breast undergoing E-SSM and E-LDMF harvesting followed by LDMF breast reconstruction 18 months after the operation. (c-d) Photographs of a 32-year-old woman 7 months after the LDMF breast reconstruction following E-SSM. (e-f) Photographs of a 36-year-old woman after E-NSM and LDMF combined with prosthesis breast reconstruction at 6 months post-operation.

**Figure 6:**
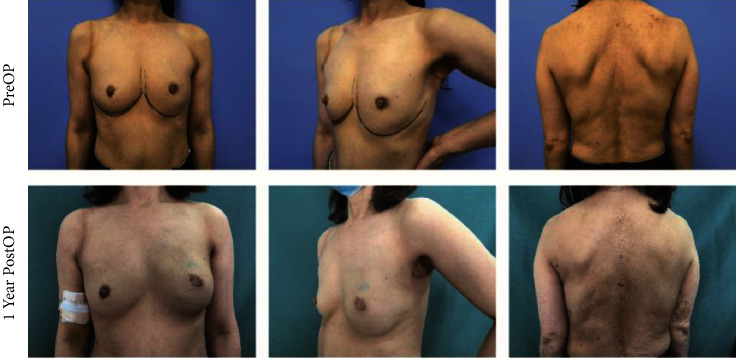
A 53-year-old woman undergoing LDMF combined with prosthesis breast reconstruction after E-SSM and E-LDMF harvesting with her preoperative photos and 1-year postoperative photos.

**Figure 7:**
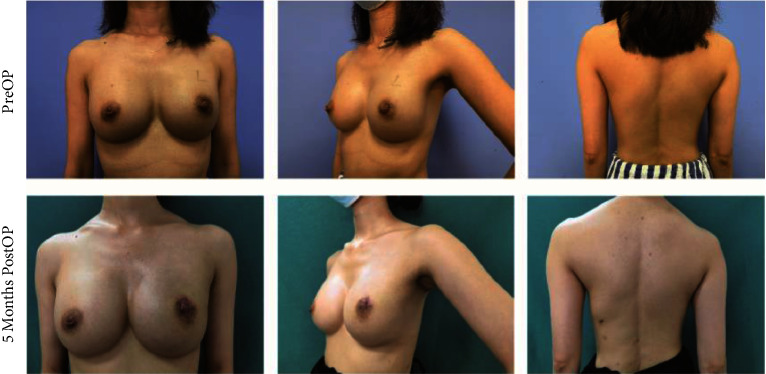
A 36-year-old woman undergoing LDMF combined with prosthesis breast reconstruction after E-SSM and E-LDMF harvesting with her preoperative photos and 5 months postoperative photos. She suffered hypopigmentation of the nipple areola.

**Figure 8:**
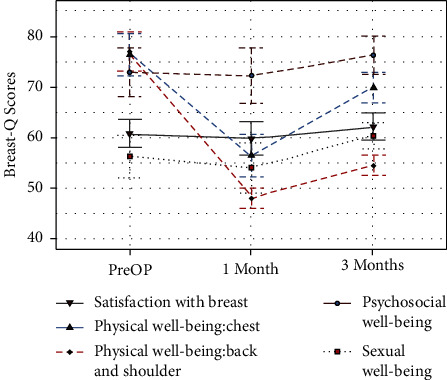
The trends of the BREAST-Q scores for breast satisfaction and quality of life before and after the operation.

The size and the characteristics of the reconstructed breast were consistent with those of the contralateral breast (see Figures 4–7). All patients completed the BREAST-Q questionnaire of preoperation and 1 month postoperation, and 2 patients have not finished the BREAST-Q questionnaire and SCAR-Q of 3 months postoperation because of the follow-up time < 3 months(Supplementary Table 1). Although they began the process of reconstruction with lower scores compared with preoperation (satisfaction with breast, *P* = 0.18; psychosocial well-being, 0.14; sexual well-being, *P* = 0.60; physical well-being: chest, *P* < 0.01; physical well-being: back and shoulder, *P* < 0.01), there was an uptrend longitudinally over time. TheBREAST-Q scores in 3 months post-operation have increased compared with 1-month post-operation. The variation trends in patient-reported outcome scores for aesthetic effects and QoL according to the BREAST-Q preoperatively and postoperatively are shown in Figure 8. There was only a scar hidden in the axillary, no scar on or around the breast and back, and patients' attitudes and feelings about the scar were good, with a mean score of 74.2 ± 8.8 on the SCAR-Q at 3 months post-operation.

## 4. Discussion

Although the LD muscle has a stable blood supply and is a reliable flap for breast reconstruction, traditional LD harvest requires a long incision that often results in poor back aesthetics and severe back trauma, significantly preventing its widespread use. Since Ramakrishnan [23] first reported endoscopic-assisted LDMF harvesting for breast reconstruction, an increasing number of endoscopic or robotic techniques for LD harvesting (Table 3) with different adaptations and disadvantages have been introduced to minimize trauma and maximize cosmetic results. After 2 years of exploration, we pioneered a novel endoscopic technique that allows the performance of NSM and LD harvesting through a single axillary approach for the treatment of breast cancer patients solving the problems of the former minimally invasive surgery (inconvenient operation, high-cost for robotic technique, long-operation time) and proved it is safe and feasible and has good cosmetic results.

Our surgical outcome—the nature and reconstructed breast without obvious incision and major complications—was higher than patients' expectations, and patients gave a good score of breast and back satisfaction and QoL at 3 months postoperatively, reaching or exceeding the scores of 6 months post-operation in another study [24]. Although our evaluation was subjective, the patients were satisfied. The only incision of this technique is hidden in the axilla, with no incisions on or around the breast and back. “Huaxi hole 2” and “Huaxi hole 2' ” are also used for postoperative drainage, thereby eliminating the need for additional incisions. In contrast, most previous studies reported that a less concealed incision [25, 26] or multiple ports [10, 14, 27, 28] or more than one incisions [11, 29] is required for endoscopic or robotic techniques to complete NSM and LD harvesting for breast reconstruction, even for partial breast reconstruction with LD [8, 16, 26]. Kim et al. [30] and Lee et al. [26] reported endoscopic-assisted LD harvesting through a 4–6 cm incision at the IMF level and on the anterior border of the LD muscle so that the scar could be hidden when lowering the arms, but another axillary incision was needed for axillary surgery and pedicle dissection or flap transfer and an incision for open breast surgery. Xu et al. [31] reported 2 cases of laparoscopic harvesting of LD flaps for breast reconstruction through three trocar ports, but their methods necessitated an incision for mastectomy. Although Liu et al. [24] reported E-LDMF harvesting using a single transverse axillary incision, several customized retractors are essential for the procedure, and the LDMF is relatively small. In 2018, Lai et al. reported one and two cases of single axillary incisions robotic-assisted NSM and LD flap harvest for IBR, but two additional trocar ports were needed, and the resected LD was only suitable for small-to-medium or partial breast reconstruction [16, 32].

Due to the thorax anatomic curvature, the narrow operative view, and the difficulty of maintaining an optical window limiting the resection of LD muscle, especially the distant LD of its paravertebral origin and iliac attachments, LD has previously only been used for smaller breast reconstruction or for volume filling after breast conservation [8], which was solved by our new endoscopic technique with the help of the “Huaxi hole 2” and the “Huaxi hole 2' ”. The inserted electric knife through these two holes acts as a baton, extending the length of the knife, overcoming the curvature of the thorax, and avoiding the chopstick effect, thus making it easier for distant LD muscle resection near its paravertebral origin or iliac bone and saving operative time. And the reverse dissection order (from deep to superficial planes) of the breast gland and LD provides adequate exposure to the operative field, greatly reducing the operation time. The E-LD muscle harvesting time decreased from a maximum of 148 min to a minimum of 56 min in the progress of repeating. In addition, it is possible to access the LD muscle near its paravertebral origin or iliac bone with the “Huaxi hole 2” enough to reconstruct a B cup breast, which meets the needs of most Asian women. Full LD or extended LD can be harvested with the additional “Huaxi hole 2′” which is suitable for breasts with moderate or greater ptosis. In our study, the longest LD muscle was 34 cm, whereas other studies have shown that LD is limited in volume and more suitable for partial or small to moderate breast reconstruction [8, 30, 32].

Another innovation of this new technique is that, in contrast to traditional surgery [8, 11, 27, 28, 33], both breast surgery and LD acquisition were performed in a reverse order (from deep to superficial planes), taking advantage of the pressure of gas inflation and the gravitational effect of the LD, thus contributing to creating the optical cavity and facilitating excision and hemostasis without the need for various specific retractors. The order of superficial LD dissection is superior, medial (lateral spine), inferior (lateral iliac spine), and lateral. We chose to dissect the anterolateral part of LAMF lastly to maximize the usefulness of the back anatomy and the tension between the LD and the skin at the lateral end, which facilitated the resection of the LD muscle. If we transected the LD muscle according to the dissection sequence of the submuscle plane, the LD muscle would retract to the lateral spine, making it difficult to resect the LD muscle. Although there is no strict sequence between LD harvesting and breast surgery, most studies performed breast surgery first, which requires more than one position change during the operation [16, 28, 30, 33]. At the beginning of this procedure, we finished the dissection from NSM to LDMF in the lateral decubitus position and then BR in the dorsal decubitus position, with a longer operation time. Later, the LD muscle was removed first in a lateral decubitus position, and then the pads under the patient's buttock and shoulder were withdrawn, naturally changing the patient's position from lateral to supine, without the need for disinfection and a towel again. In addition, our homemade access to endoscopic instruments is much cheaper, more flexible, and better-functioning. It partly overcomes the disadvantages of limited internal mobility and inadequate dissection angles because trocars are not restricted by soft gloves and protector.

Postoperative complications are a concern for surgeons. Minimally invasive techniques offer the greatest potential advantage in terms of the complication of flap ischemia, as there are no incisions on or around the breast and back, and there is less impact on the blood supply to the flap. Moreover, the image magnification of the endoscopic technique allows for greater clarity of the operative area, facilitating the identification and protection of blood vessels. In our study, only one patient suffered hypopigmentation of the nipple areola, and no patient experienced ischemic or necrosis of breast skin, donor-site flap, or NAC. Although autologous breast reconstruction may increase donor-site complications, endoscopic or robotic techniques have greatly decreased these complications. Because back seroma is more common in LD breast reconstruction [8, 26, 34], pressure dressing was used routinely over the back, negative pressure was continuously applied to induce drainage of the back, stricter criteria for extubation (less than 20 ml per day for 3 consecutive days) were complied with, and excessive upper extremity use was restricted. If a back seroma has developed, an inactivated *Pseudomonas aeruginosa* preparation would be injected into the operation area of the back to trigger a tissue repair response, causing a strong local sterile inflammatory reaction and promoting the adherence and fibrosis of the skin and subcutaneous region of the back. A total of 4 patients in our study developed donor-site seroma, which was conservatively managed.

However, due to the small sample size and short follow-up period in this study, more studies are needed, and further validation of the above findings is expected.

## 5. Conclusion

In conclusion, this original and novel endoscopic technique for NSM and LD harvesting for breast reconstruction through a single axillary approach is safe, feasible, and has good cosmetic results. It has a wide scope of application in patients who meet the indications and can be promoted as a routine procedure.

## Figures and Tables

**Table 1:** Clinical characteristics of the patients.

Parameter	Mean ± SD or No. (%)
Age, years	38.7 ± 7.5
Median follow-up period, range, months	7.5 (1–23)
BMI (kg/m^2^)	21.3 ± 2.7
Active tobacco use	0 (0)
Diabetes	0 (0)

Tumor location	
Left	7 (46.7)
Right	8 (53.3)

Pathological types	
Invasive carcinoma	10 (50)
Carcinoma in situ	9 (45)
Other type	1 (5)

*T*	
Tis	5 (33.3)
T1	5 (25.0)
T2	9 (5.0)
T3^a^	1 (5.0)

*N*	
N0	16 (80.0)
N1	4 (20.0)

Cup size	
A-B	10 (50.0)
C	7 (35.0)
>C	3 (15.0)

Degree of breast ptosis^b^	
I	7 (35.0)
II	4 (20.0)
Neoadjuvant chemotherapy	4 (20.0)

*Abbreviations*. BMI: body mass index; SD: standard deviation. ^a^ The 1 large unicentric carcinoma shrank to <5 cm after neoadjuvant chemotherapy. ^b^ Regnault breast ptosis grade, 9 patients with no breast ptosis.

**Table 2:** Operative and perioperative data.

Characteristic	No. (%)
Mastectomy weight, mean ± SD (g)	278.3 ± 82.3
Axillary surgery^a^	
SLNB only	12 (60.0)
ALND	5 (25.0)
SLNB then ALND	1 (5.0)

Reconstruction surgery	
LDMF	5 (25.0)
LDMF + prothesis	15 (75.0)
Total operation time, mean ± SD (min)	262.6 ± 54.4
LDMF harvesting time, mean ± SD (min)	96.5 ± 25.3
NSM time, mean ± SD (min)	56.0 ± 14.7

LDMF size, mean ± SD (cm)	
Length	26.9 ± 3.1
Width	13.8 ± 2.1
Implant volume (cc)	125–480
Intraoperative bleeding (ml)	48.5 ± 14.2
Hospital stays, mean ± SD, days	7.4 ± 1.7

Nipple management^b^	
NSM	14 (70.0)
SSM without nipple reconstruction	1 (5.0)
SSM with nipple reconstruction	4 (20.0)

Hospitalization expenses, mean ± SD, USD	
LDMF	3401.6 ± 457.3
LDMF ± prothesis	5434.8 ± 609.2

SD: standard deviation; SLNB: sentinel lymph node biopsy; ALND: axillary lymph node dissection; NSM: nipple-sparing mastectomy; SSM: skin-sparing mastectomy; LDMF: latissimus dorsi muscle flap; USD: United States dollar. ^a^ There were two patients with no axillary surgery. One patient with multiple tumors was diagnosed with benign tumors with intraoperative frozen section; another patient had undergone traditional NSM, ALND, and expander breast reconstruction previously. ^b^ One patient had undergone NSM. Subcutaneous and muscular dissection is performed to enter the pocket and remove the tissue expander in this surgery.

**Table 3:** Surgical data (various authors).

Author (references)	Patients	Mastectomy type	Incision length (cm) and situation	Other incisions or ports	Endoscopy or robot	LD harvesting time (min)
Winocour et al. [10]	25	Mastectomy	3 ports, anterior border of the muscle, and 7, 14, and 21 cm from the posterior axillary line	Mastectomy incision	Endoscopy	—
Lee et al. [26]	5	Partial mastectomy	4–6, in the mid-axillary line at the IMF level	Axillary incision, breast incision	Endoscopy	82.6 (65–95)
Kim et al. [30]	21	NSM, partial mastectomy	4, at the IMF level and on the anterior border of the LD muscle	Axillary incision, breast incision	Endoscopy	63 (40–121)
Lai et al. [16]	1	Quadrantectomy, robot	Axillary incision	2 ports	Robot	97
Lai et al. [32]	2	NSM, robot	Axillary incision	2 ports	Robot	267 and 90
Xu et al. [31]	2	Lumpectomy	3 ports	Breast incision	Endoscopy	161 and 180
Houvenaeghel et al. [27]	46	SSM	NAC incision, 6-7 cm vertical incision at the level of the mid-axillary line (not necessary)	3 ports	Robot	—

## Data Availability

The data used to support the findings of this study are available from the corresponding author upon request.

## Additional Points

*Reporting Checklist.* The authors have completed the STROBE reporting checklist. See Supplementary.

## Supplementary Materials

Supplementary Materials Video 1: Surgical procedures of immediate breast reconstruction with latissimus dorsi flap of breast cancer patients after Nipple-sparing mastectomy under endoscope.

## References

[1] Chen W., Zheng R., Baade P. D. (2016). Cancer statistics in China, 2015. *CA: A Cancer Journal for Clinicians*.

[2] Allemani C., Matsuda T., Di Carlo V. (2018). Global surveillance of trends in cancer survival 2000-14 (CONCORD-3): analysis of individual records for 37 513 025 patients diagnosed with one of 18 cancers from 322 population-based registries in 71 countries. *The Lancet*.

[3] Jia-Jian C., Nai-Si H., Jing-Yan X. (2015). Current status of breast reconstruction in southern China: a 15 Year, single institutional experience of 20, 551 breast cancer patients. *Medicine (Baltimore)*.

[4] Mandelbaum A. D., Thompson C. K., Attai D. J. (2020). National trends in immediate breast reconstruction: an analysis of implant-based versus autologous reconstruction after mastectomy. *Annals of Surgical Oncology*.

[5] Sood R., Easow J. M., Konopka G., Panthaki Z. J. (2018). Latissimus dorsi flap in breast reconstruction: recent innovations in the workhorse flap. *Cancer Control*.

[6] Adams W. P., Lipschitz A. H., Ansari M., Kenkel J. M., Rohrich R. J. (2004). Functional donor site morbidity following latissimus dorsi muscle flap transfer. *Annals of Plastic Surgery*.

[7] Marin-Gutzke M., Sanchez-Olaso A. (2010). Reconstructive surgery in young women with breast cancer. *Breast Cancer Research and Treatment*.

[8] Yang C. E., Roh T. S., Yun I. S., Kim Y. S., Lew D. H. (2014). Immediate partial breast reconstruction with endoscopic latissimus dorsi muscle flap harvest. *Archives of Plastic Surgery*.

[9] Berthet G., Faure C., Dammacco M. A. (2018). Tolerance of latissimus dorsi in immediate breast reconstruction without implant to radiotherapy. *Journal of Plastic, Reconstructive & Aesthetic Surgery*.

[10] Winocour S., Tarassoli S., Chu C. K., Liu J., Clemens M. W., Selber J. C. (2020). Comparing outcomes of robotically assisted latissimus dorsi harvest to the traditional open approach in breast reconstruction. *Plastic and Reconstructive Surgery*.

[11] Ma J. X., Li B., Xia Y. C. (2022). Latissimus dorsi muscle flap transfer through endoscopic approach combined with the implant after tissue expansion for breast reconstruction of mastectomy patients. *BMC Surgery*.

[12] Lai H. W., Chen S. T., Tai C. M. (2020). Robotic- versus endoscopic-assisted nipple-sparing mastectomy with immediate prosthesis breast reconstruction in the management of breast cancer: a case-control comparison study with analysis of clinical outcomes, learning curve, patient-reported aesthetic results, and medical cost. *Annals of Surgical Oncology*.

[13] Toesca A., Peradze N., Galimberti V. (2017). Robotic nipple-sparing mastectomy and immediate breast reconstruction with implant: first report of surgical technique. *Annals of Surgery*.

[14] Chung J. H., You H. J., Kim H. S., Lee B. I., Park S. H., Yoon E. S. (2015). A novel technique for robot assisted latissimus dorsi flap harvest. *Journal of Plastic, Reconstructive & Aesthetic Surgery*.

[15] Leonardis J. M., Diefenbach B. J., Lyons D. A. (2019). The influence of reconstruction choice and inclusion of radiation therapy on functional shoulder biomechanics in women undergoing mastectomy for breast cancer. *Breast Cancer Research and Treatment*.

[16] Lai H. W., Chen S. T., Lin S. L. (2018). Technique for single axillary incision robotic assisted quadrantectomy and immediate partial breast reconstruction with robotic latissimus dorsi flap harvest for breast cancer: a case report. *Medicine (Baltimore)*.

[17] Liang F., Wen N., Xie Y. (2021). Subversion of endoscopic breast reconstruction Surgery “nipple-sparing mastectomy and immediate reconstruction with a latissimus dorsi flap/latissimus dorsi flap and implant through a single axillary incision”. *Annals of Surgery Open*.

[18] Zhou J., Liu X., Feng Y. (2021). Breakthrough in breast reconstruction in the context of COVID-19: safety and efficiency of endoscopic breast reconstruction at a day surgery center. *Gland Surgery*.

[19] Zhang S., Xie Y., Liang F. (2021). Video-assisted transaxillary nipple-sparing mastectomy and immediate implant-based breast reconstruction: a novel and promising method. *Aesthetic Plastic Surgery*.

[20] Pusic A. L., Klassen A. F., Scott A. M., Klok J. A., Cordeiro P. G., Cano S. J. (2009). Development of a new patient-reported outcome measure for breast surgery: the BREAST-Q. *Plastic and Reconstructive Surgery*.

[21] Marquez J. E., Kapadia K., Ghosh K., Silvestri B., Singh G., Huston T. L. (2020). Efficacy of fibrin sealants in preventing seroma formation in reduction mammaplasty: a single surgeon’s experience. *Annals of Plastic Surgery*.

[22] Srivastava V., Basu S., Shukla V. K. (2012). Seroma formation after breast cancer surgery: what we have learned in the last two decades. *Journal of Breast Cancer*.

[23] Ramakrishnan V., Southern S. J., Tzafetta R. (2000). Reconstruction of the high-risk chest wall with endoscopically assisted latissimus dorsi harvest and expander placement. *Annals of Plastic Surgery*.

[24] Liu C., Luan J., Ouyang Y. (2019). Breast reconstruction in Poland syndrome patients with latissimus dorsi myo flap and implant: an efficient endoscopic approach using single transverse axillary incision. *Aesthetic Plastic Surgery*.

[25] Yuan H., Xie D., Xiao X., Huang X. (2017). The clinical application of mastectomy with single incision followed by immediate laparoscopic-assisted breast reconstruction with latissimus dorsi muscle flap. *Surgical Innovation*.

[26] Lee J., Jung J. H., Kim W. W., Park C. S., Lee R. K., Park H. Y. (2020). Endoscopy-assisted muscle-sparing Latissimus Dorsi muscle flap harvesting for partial breast reconstruction. *BMC Surgery*.

[27] Houvenaeghel G., El Hajj H., Schmitt A. (2020). Robotic-assisted skin sparing mastectomy and immediate reconstruction using latissimus dorsi flap a new effective and safe technique: a comparative study. *Surgical Oncology*.

[28] Chang H. P., Fan K. L., Song S. Y., Lee D. W. (2020). The traditional versus endoscopic-assisted latissimus dorsi harvest in oncoplastic surgery: a long term comparison of breast volume, aesthetics, and donor site outcomes. *Asian Journal of Surgery*.

[29] Missana M. C., Pomel C. (2007). Endoscopic latissimus dorsi flap harvesting. *The American Journal of Surgery*.

[30] Kim D. G., Kim J. S., Lee J. S., Lee J., Park H. Y., Yang J. D. (2021). The usefulness of endoscopic harvesting of the latissimus dorsi flap for breast reconstruction using a single-port and CO2 gas insufflation technique. *Aesthetic Plastic Surgery*.

[31] Xu S., Tang P., Chen X. (2016). Novel technique for laparoscopic harvesting of latissimus dorsi flap with prosthesis implantation for breast reconstruction: a preliminary study with 2 case reports. *Medicine (Baltimore)*.

[32] Lai H. W., Lin S. L., Chen S. T. (2018). Robotic nipple sparing mastectomy and immediate breast reconstruction with robotic latissimus dorsi flap harvest - technique and preliminary results. *Journal of Plastic, Reconstructive & Aesthetic Surgery*.

[33] Iglesias M., Gonzalez-Chapa D. R. (2013). Endoscopic latissimus dorsi muscle flap for breast reconstruction after skin-sparing total mastectomy: report of 14 cases. *Aesthetic Plastic Surgery*.

[34] Clough K. B., Louis-Sylvestre C., Fitoussi A., Couturaud B., Nos C. (2002). Donor site sequelae after autologous breast reconstruction with an extended latissimus dorsi flap. *Plastic and Reconstructive Surgery*.

