# Supplementary material for: Endoscopic Nipple- or Skin-Sparing Mastectomy and Immediate Breast Reconstruction with Endoscopic Harvesting of the Latissimus Dorsi Flap: A Preliminary Experience of an Innovative Technique
Source: Breast J. 2022 Oct 27;2022:1373899. doi: 10.1155/2022/1373899 (PMC9633195; doi:10.1155/2022/1373899)
Supplement: Supplementary Materials — Video 1: Surgical procedures of immediate breast reconstruction with latissimus dorsi flap of breast cancer patients after Nipple-sparing mastectomy under endoscope. [file 1373899.f1.zip › 1373899.f1.docx]

**Supplementary Table 1** BREAST-Q domains and scores

| BREAST-Q Dimensions | Preoperation | 1 month post-operation | 3 months post-operation | F value | P value |
| --- | --- | --- | --- | --- | --- |
| Satisfaction with breast | 65.3±12.2 | 59.8±15.0 | 62.2±12.2 | 1.59 | 0.23 |
| Satisfaction with back^a^ | - | 60.0±20.1 | 77.2±11.4 | - | 0.05 |
| Psychosocial well-being | 72.9±21.8 | 71.2±24.5 | 76.3±16.8 | 0.52 | 0.60 |
| Sexual well-being | 56.3±18.8 | 54.0±22.2 | 60.3±11.6 | 1.18 | 0.32 |
| Physical well-being: chest | 76.5±18.8 | 56.4±18.9 | 69.9±14.2 | 10.45 | <0.01 |
| Physical well-being: back and shoulder | 77.1±17.6 | 48.0±9.3 | 54.4±9.3 | 33.93 | <0.01 |

^a^There is no preoperative scale of the BREAST-Q™ Reconstruction Module questionnaire

STROBE Statement—Checklist of items that should be included in reports of ***cohort studies***

|  | Item No | Recommendation | Reported on Page Number/Line Number | Reported on Section/Paragraph |
| --- | --- | --- | --- | --- |
| **Title and abstract** | 1 | (*a*) Indicate the study’s design with a commonly used term in the title or the abstract | Page2/line 30-36 | Abstract/Paragraph2 |
|  |  | (*b*) Provide in the abstract an informative and balanced summary of what was done and what was found | Page2/line 36-51 | Abstract/Paragraph2-3 |
| Introduction | | |  |  |
| Background  /rationale | 2 | Explain the scientific background and rationale for the investigation being reported | Page3/line59-Page4/line 98 | Introduction/Paragraph1-2 |
| Objectives | 3 | State specific objectives, including any prespecified hypotheses | Page4/line 99-109 | Introduction/Paragraph3 |
| Methods | | |  |  |
| Study design | 4 | Present key elements of study design early in the paper | Page4/line 114-120 | Patients/Paragraph1 |
| Setting | 5 | Describe the setting, locations, and relevant dates, including periods of recruitment, exposure, follow-up, and data collection | Page4/line 114-116 | Patients/Paragraph1 |
| Participants | 6 | (*a*) Give the eligibility criteria, and the sources and methods of selection of participants. Describe methods of follow-up | Page5/line 121-132 and Page8/line 200-208 | Patients/Paragraph2-3 and Postoperative management/Paragraph1 |
|  |  | (*b*) For matched studies, give matching criteria and number of exposed and unexposed | NA | Not involved |
| Variables | 7 | Clearly define all outcomes, exposures, predictors, potential confounders, and effect modifiers. Give diagnostic criteria, if applicable | Page8/line 210-Page9/line 232 | Outcome measures evaluation/Paragraph1-2 |
| Data sources/ measurement | 8* | For each variable of interest, give sources of data and details of methods of assessment (measurement). Describe comparability of assessment methods if there is more than one group | Page8/line 210-Page9/line 232 | Outcome measures evaluation/Paragraph1-2 |
| Bias | 9 | Describe any efforts to address potential sources of bias | NA | Not involved |
| Study size | 10 | Explain how the study size was arrived at | Page5/line 119-120 | Patients/Paragraph1 |
| Quantitative variables | 11 | Explain how quantitative variables were handled in the analyses. If applicable, describe which groupings were chosen and why | NA | Not involved |
| Statistical methods | 12 | (*a*) Describe all statistical methods, including those used to control for confounding | Page9/line 234-240 | Statistical analysis/Paragraph1 |
|  |  | (*b*) Describe any methods used to examine subgroups and interactions | NA | Not involved |
|  |  | (*c*) Explain how missing data were addressed | NA | Not involved |
|  |  | (*d*) If applicable, explain how loss to follow-up was addressed | NA | Not involved |
|  |  | (*e*) Describe any sensitivity analyses | NA | Not involved |
| Results | | |  |  |
| Participants | 13* | (a) Report numbers of individuals at each stage of study—eg numbers potentially eligible, examined for eligibility, confirmed eligible, included in the study, completing follow-up, and analysed | Page9/line 243-Page10/line 249 | Clinical and pathological characteristics/Paragraph1 |
|  |  | (b) Give reasons for non-participation at each stage | NA | Not involved |
|  |  | (c) Consider use of a flow diagram | NA | Not involved |
| Descriptive data | 14* | (a) Give characteristics of study participants (eg demographic, clinical, social) and information on exposures and potential confounders | Page9/line 243-Page10/line 249 | Clinical and pathological characteristics/Paragraph1 |
|  |  | (b) Indicate number of participants with missing data for each variable of interest | Page9/line 243-Page10/line 249 | Clinical and pathological characteristics/Paragraph1 |
|  |  | (c) Summarise follow-up time (eg, average and total amount) | Page10/line 266 | Complications/Paragraph1 |
| Outcome data | 15* | Report numbers of outcome events or summary measures over time | Page10/line 266- Page11/line 285 | Complications/Paragraph1; Aesthetic results and QoL/Paragraph1 |
| Main results | 16 | (*a*) Give unadjusted estimates and, if applicable, confounder-adjusted estimates and their precision (eg, 95% confidence interval). Make clear which confounders were adjusted for and why they were included | NA | Not involved |
|  |  | (*b*) Report category boundaries when continuous variables were categorized | NA | Not involved |
|  |  | (*c*) If relevant, consider translating estimates of relative risk into absolute risk for a meaningful time period | NA | Not involved |
| Other analyses | 17 | Report other analyses done—eg analyses of subgroups and interactions, and sensitivity analyses | NA | Not involved |
| Discussion | | |  |  |
| Key results | 18 | Summarise key results with reference to study objectives | Page11/line 289- Page12/line 298 | Discussion/Paragraph1 |
| Limitations | 19 | Discuss limitations of the study, taking into account sources of potential bias or imprecision. Discuss both direction and magnitude of any potential bias | Page14/line 375-376 | Discussion/Paragraph6 |
| Interpretation | 20 | Give a cautious overall interpretation of results considering objectives, limitations, multiplicity of analyses, results from similar studies, and other relevant evidence | Page12/line 298- Page14/line 374 | Discussion/Paragraph1-5 |
| Generalisability | 21 | Discuss the generalisability (external validity) of the study results | Page14/line 377-380 | Conclusion/Paragraph1 |
| Other information | | |  |  |
| Funding | 22 | Give the source of funding and the role of the funders for the present study and, if applicable, for the original study on which the present article is based | Page21/line 418 | Funding/Paragraph1 |

*Give information separately for exposed and unexposed groups.

**Note:** An Explanation and Elaboration article discusses each checklist item and gives methodological background and published examples of transparent reporting. The STROBE checklist is best used in conjunction with this article (freely available on the Web sites of PLoS Medicine at http://www.plosmedicine.org/, Annals of Internal Medicine at http://www.annals.org/, and Epidemiology at http://www.epidem.com/). Information on the STROBE Initiative is available at http://www.strobe-statement.org.
